# Supplementary material for: Recellularized Colorectal Cancer Patient-Derived Scaffolds as In Vitro Pre-Clinical 3D Model for Drug Screening
Source: Cancers (Basel). 2020 Mar 13;12(3):681. doi: 10.3390/cancers12030681 (PMC7140024; doi:10.3390/cancers12030681)
Supplement: Supplementary file 1 [file cancers-12-00681-s001.pdf]

Recellularized colorectal cancer patient-derived scaffolds as in vitro pre-clinical 3D model for drug screening

Francesca Sensi[1,2], Edoardo D'Angelo[3,4], Martina Piccoli[1], Piero Pavan[5], Francesca Mastrotto[6], Paolo Caliceti[6], Andrea Biccari[4], Diana Corallo[1], Luca Urbani[7,8], Matteo Fassan[9], Gaya Spolverato[3], Pietro Riello[2], Salvatore Pucciarelli[3], Marco Agostini[1,3,4,*]

[1] Fondazione Istituto di Ricerca Pediatrica Città della Speranza, Padua, Italy
[2] Department of Molecular Sciences and Nanosystems, Ca' Foscari University of Venice, Italy.
[3] First Surgical Clinic, Department of Surgery, Oncology and Gastroenterology, University of Padua, Italy
[4] LIFELAB Program, Consorzio per la Ricerca Sanitaria-CORIS, Veneto Region, Padua, Italy
[5] Department of Industrial Engineering, University of Padua, Italy
[6] Department of Pharmaceutical and Pharmacological Sciences, University of Padua, Italy
[7] Institute of Hepatology, Foundation for Liver Research, London, UK
[8] Faculty of Life Sciences & Medicine, King's College London, UK
[9] Surgical Pathology Unit, Department of Medicine, University of Padua, Italy.

*Correspondence: m.agostini@unipd.it ; Tel: +39 049 9640160 ; Fax: +39 049 9640127

Supplementary figures and tables

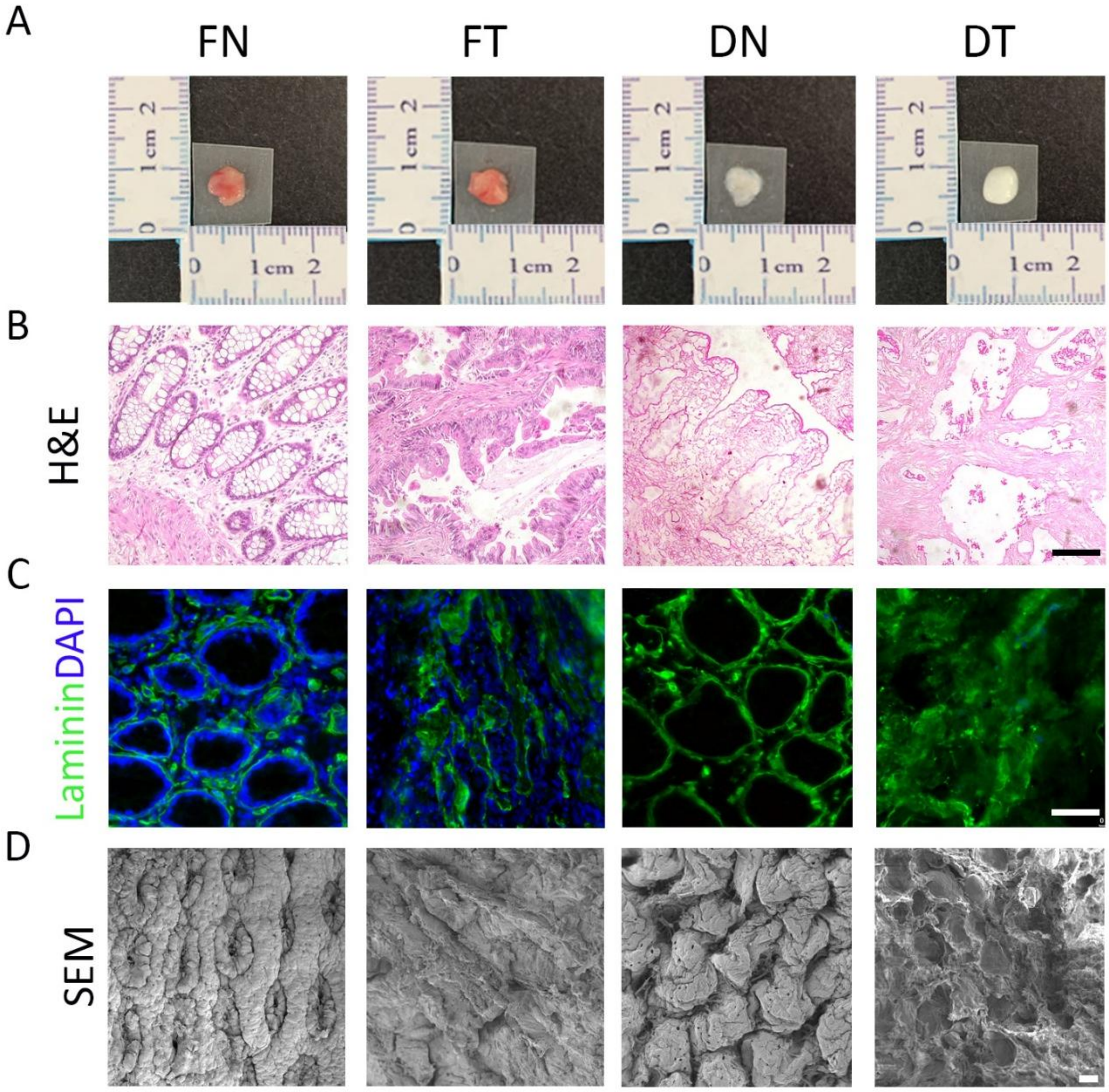

Supplementary figure 1: Decellularization and characterization of patient-derived decellularized scaffolds of normal healthy mucosa and CRC. **(A)** Gross appearance of fresh (FN and FT) and decellularized (DN and DT) biopsies before and after two detergent enzymatic cycles. **(B)** Haematoxylin and Eosin (H&E) histological staining of fresh and decellularized biopsies (scale bar = 75 µm). **(C)** Immunofluorescence of Laminin (green) in fresh and decellularized samples. Nuclei are counterstained with DAPI (blue); (scale bar = 50 µm). **(D)** Scanning electron microscopy (SEM) of fresh and decellularized biopsies; (scale bar = 20 µm).

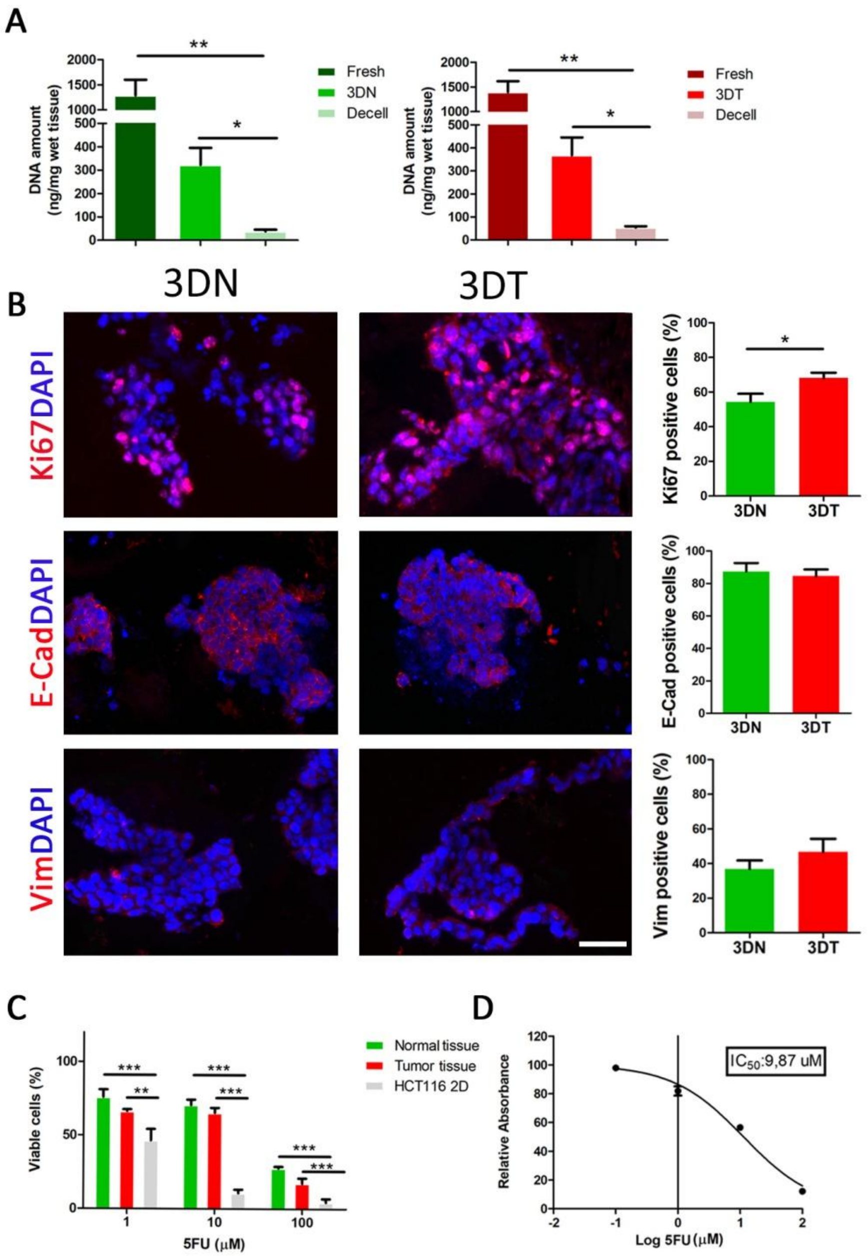

Supplementary figure 2: Characterization of matched HCT116 recellularized samples from 3DN and 3DT specimens. **(A)** immunofluorescence stainings in 3DT and 3DN and quantifications: Ki67, as proliferation marker; E-cadherin as epithelial marker; Vimentin, as mesenchymal marker a; Laminin to highlight basement membrane structure; DAPI to counterstain nuclei (scale bar = 100 µm). **(B)** DNA amount quantification in fresh samples, after decellularization process and after 5 days of repopulation with HCT116, in both 3DN and 3DT. **(C)** Comparison between percentages of viable cells (by absorbance fold change detection) after administration of 5FU at 1-10-100 µM in a 2D culture and in both 3DN and 3DT models. **(D)** Calculation of 5FU 3D IC_{50} by nonlinear regression (*: p-value < .05; **: p-value < .01; ***: p-value < .001).

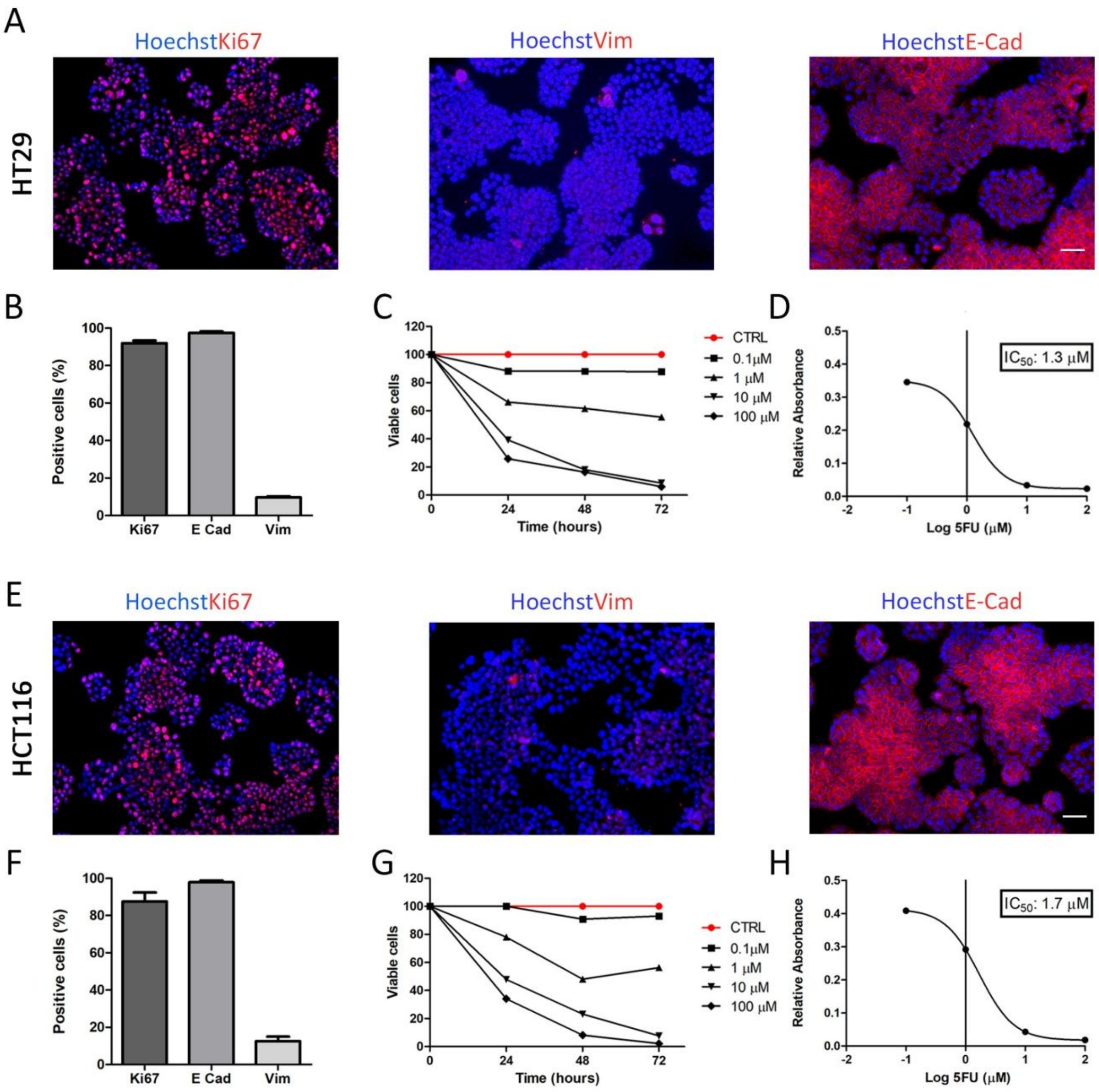

Supplementary figure 3: Characterization of HT29 and HCT116 cells growing in 2D. **(A-E)** Immunofluorescence staining in HT29 and HCT116 cells: Ki67, as proliferation marker; E-cadherin as epithelial marker; Vimentin, as mesenchymal marker and Hoechst to counterstain nuclei (scale bar = 100 µm). **(B-F)** Quantification of positive cells (%) for markers Ki67, E-cadherin and Vimentin in HT29 and HCT116 cells. **(C-G)** Evaluation of drug sensitivity to 5FU in HT29 and HCT116 2D cultured cells using absorbance fold change detection (indicating cell viability). **(D-H)** 5FU HT29 and HCT116 IC_{50} calculation by nonlinear regression.

	Tissue permeability K (mm^4/Ns)				
**Sample #**	**FN**	**FT**	**DN**	**DT**	**3DT**
1	1.6	96.5	43.3	1189.4	295.8
2	10.5	148.0	16.1	3102.4	360.3
3	17.3	36.1	281.3	6323.6	200.1
4	-	-	2582.1	3887.2	-
**Average**	9.8	93.5	917.9	3625.7	285.4
**SD**	4.1	55.9	1048.3	1430.3	80.6

Supplementary table 1: Estimated values of permeability on the different types of tissue. FN: Fresh Normal colon, FT: Fresh Tumor colon, DN: Decellularized Normal colon, DT: Decellularized Tumor colon, 3DT: Recellularized Tumor colon (SD: standard deviation).
